# Co-application of biochar and compost enhanced soil carbon sequestration in urban green space

**DOI:** 10.3389/fmicb.2025.1707894

**Published:** 2025-11-06

**Authors:** Ben Wang, Wenfang Li, Na Xue, Ruyang Xi, Yanjun Wang, Lele Fang, Qiang Wang, Xinchun Liang, Yiqian Xiao, Xiuyun Yang, Xiaogang Wu

**Affiliations:** 1 College of Forestry, Shanxi Agricultural University, Taigu, Shanxi, China; 2 College of Urban and Rural Construction, Shanxi Agricultural University, Taigu, Shanxi, China; 3 College of Horticulture, Shanxi Agricultural University, Taigu, China; 4 Shanxi Xiyu Expressway Co., Ltd., Jinzhong, Shanxi, China; 5 China Highway Engineering Consulting Corporation, Beijing, China

**Keywords:** urban green space, biochar, compost, metagenomics, soil carbon sequestration

## Abstract

The mechanism of biochar and compost as soil amendments in urban green spaces remains unclear. Using *Euonymus kiautschovicus* as a model system, this study established eight treatment gradients, 0 (CK), single biochar applications: 4% (BC4), 8% (BC8), 12% (BC12), 7.5% compost (COM), and their combinations BCC4 (BC4 + 7.5% COM), BCC8 (BC8 + 7.5% COM), BCC12 (BC12 + 7.5% COM). Through metagenomic sequencing and metagenome-assembled genomes (MAGs) analysis, we investigated soil microbiome structure, carbon sequestration functional genes, and their interactions in response to amendments. The combined application of medium-low dose biochar (4–8%) with compost significantly optimized the physicochemical properties and microbial functions in soils. Compared to single amendments, hybrid treatments synergistically enhanced soil moisture content. Specifically, BCC8 increased by 27% compared to the CK, organic carbon levels reached 12.8 g/kg with BCC12, and available nutrients showed 45% higher available phosphorus with BCC4. Metagenomic analysis revealed that hybrid treatments reshaped microbial community structure, with BCC8 significantly enriching *Acidobacteria* (8.72%) and *Nitrospira* (1.42%), driving an increased abundance of carbon fixation genes. Among key carbon fixation pathways, the reductive tricarboxylic acid cycle (rTCA) exhibited the highest gene abundance (mean 15.03), dominated by MAG176. The Calvin-Benson-Bassham (CBB) cycle displayed broad adaptability, with MAG59 identified as a core carbon-fixing strain. This study has significant implications for the application of biochar-compost combinations in carbon management of urban green spaces.

## Graphical abstract

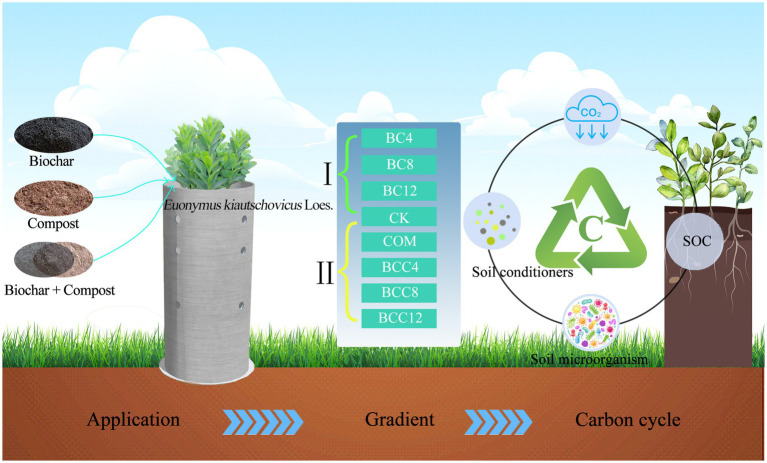

## 1 Introduction

Soils around the world are undergoing varying degrees of degradation, with the carbon sink function of urban green spaces continuously weakening or even disappearing (Xie et al., 2019; Vieillard et al., 2024). Urban green space can purify the environment, regulate the climate, maintain biodiversity, and play a vital role in improving the urban ecological environment. As the foundation of the green space ecosystem functions, soils provide critical ecosystem services (Zhu et al., 2017). Urban green spaces, serving as vital natural carbon sinks in cities, have seen their carbon sequestration potential enhancement emerge as a key strategy for low-carbon urban development (Guo et al., 2024). Biochar and compost, as sustainable soil amendments, have become global research hotspots for soil carbon sequestration and microbial regulation due to their unique physicochemical properties and ecological functions (Barrow, 2012; Mohd et al., 2013; Barthod et al., 2018). Biochar, formed through high-temperature pyrolysis of biomass, features a highly aromatic structure, large specific surface area, and high C/N ratio, enhancing soil carbon stability (Herath et al., 2013; Lehmann et al., 2015; Smith, 2016). Compost, rich in labile organic matter and functional microbial communities, directly promotes soil nutrient cycling (Laird et al., 2010; Nobile et al., 2022). Their combined application in agricultural soils has demonstrated synergistic improvements in soil structure, enhanced microbial activity, and increased carbon sequestration potential (Han et al., 2023; Iswahyudi et al., 2024). However, urban green space soils exhibit unique physicochemical characteristics such as high compaction, low organic matter content, and heterogeneous microbial communities (Scharenbroch et al., 2005), resulting in fundamentally distinct soil amendment mechanisms compared to agricultural systems (Edmondson et al., 2014). Elucidating the microbiome responses and carbon fixation gene regulatory mechanisms of biochar and compost in urban garden soils holds significant scientific importance for optimizing urban carbon sink management strategies.

Biochar addition enhances microbial ecological niches by increasing porosity and adsorption capacity (Dahlawi et al., 2018), while compost inputs stimulate microbial metabolic activity through dissolved organic carbon release (Mehta et al., 2014; Rastogi et al., 2020; Palaniveloo et al., 2020). Metagenomic analyses reveal positive correlations between biochar-induced Actinobacteria enrichment and carbon fixation gene expression, alongside increased abundances of Bacteroidetes and Acidobacteria but decreased Proteobacteria (Kolton et al., 2011; Anderson et al., 2011). Compost-driven Proteobacteria proliferation may indirectly influence carbon cycling through enhanced polysaccharide degradation genes (Yuan et al., 2015; Czekała et al., 2016; Yang et al., 2019). Combined biochar-compost applications demonstrate greater efficacy in carbon stabilization and sequestration compared to biochar alone (Jien et al., 2015). While synergistic effects of biochar-compost combinations have been observed in agricultural soils and turfgrass systems (Wang et al., 2017; Hale et al., 2021), with biochar mitigating compost-induced carbon mineralization losses (Yang et al., 2025), these phenomena remain poorly characterized in urban soils. Post-application of biochar and compost significantly enhances soil nitrogen fixation, alters microbial community structure, and effectively reduces potentially phytotoxic PAH (Polynuclear Aromatic Hydrocarbons‌) in biochar (Li et al., 2020; Liu X. et al., 2022). However, research on the carbon sequestration mechanisms of biochar-compost combinations in urban green space soils remains limited, with most studies focusing on single amendments or low-dose treatments (typically <5%) (Lehmann et al., 2011; Liu et al., 2016; Xu et al., 2021). Systematic evaluations of high-dose biochar gradients (e.g., >5%) and biochar-compost interactions remain insufficient (Sulemana et al., 2021).

While significant progress has been made in biochar and compost research (Agegnehu et al., 2015; Liang M. et al., 2021), key knowledge gaps persist: (1) What is the response pattern of the soil microbiome to biochar/compost in urban green space? (2) Could high-dose biochar combined with compost induce inhibitory effects or novel synergistic mechanisms? (3) Existing studies predominantly rely on 16S rRNA sequencing or single functional gene detection (Yang et al., 2024; Awasthi et al., 2017), lacking multidimensional metagenomic correlation analyses.

This study employs *E. kiautschovicus* as a model plant, establishing eight gradient treatments to investigate the effects of different biochar and compost dosages on urban potted soil microbial community structure, network stability, key carbon fixation gene abundance dynamics, driving factors, and the coupling mechanisms between microbial functional modules and soil carbon pool formation. MAGs technology is utilized to resolve functional gene-host microbe relationships and reveal urban green space-specific microbial response patterns, providing a theoretical foundation for developing precision carbon management strategies. The results of this study will help bridge the knowledge gap in the microbial mechanisms of soil amendments of urban green space, promoting the practical application of ecological development in line with the carbon neutrality target.

## 2 Materials and methods

### 2.1 Study area and plant species

**Figure 1:**
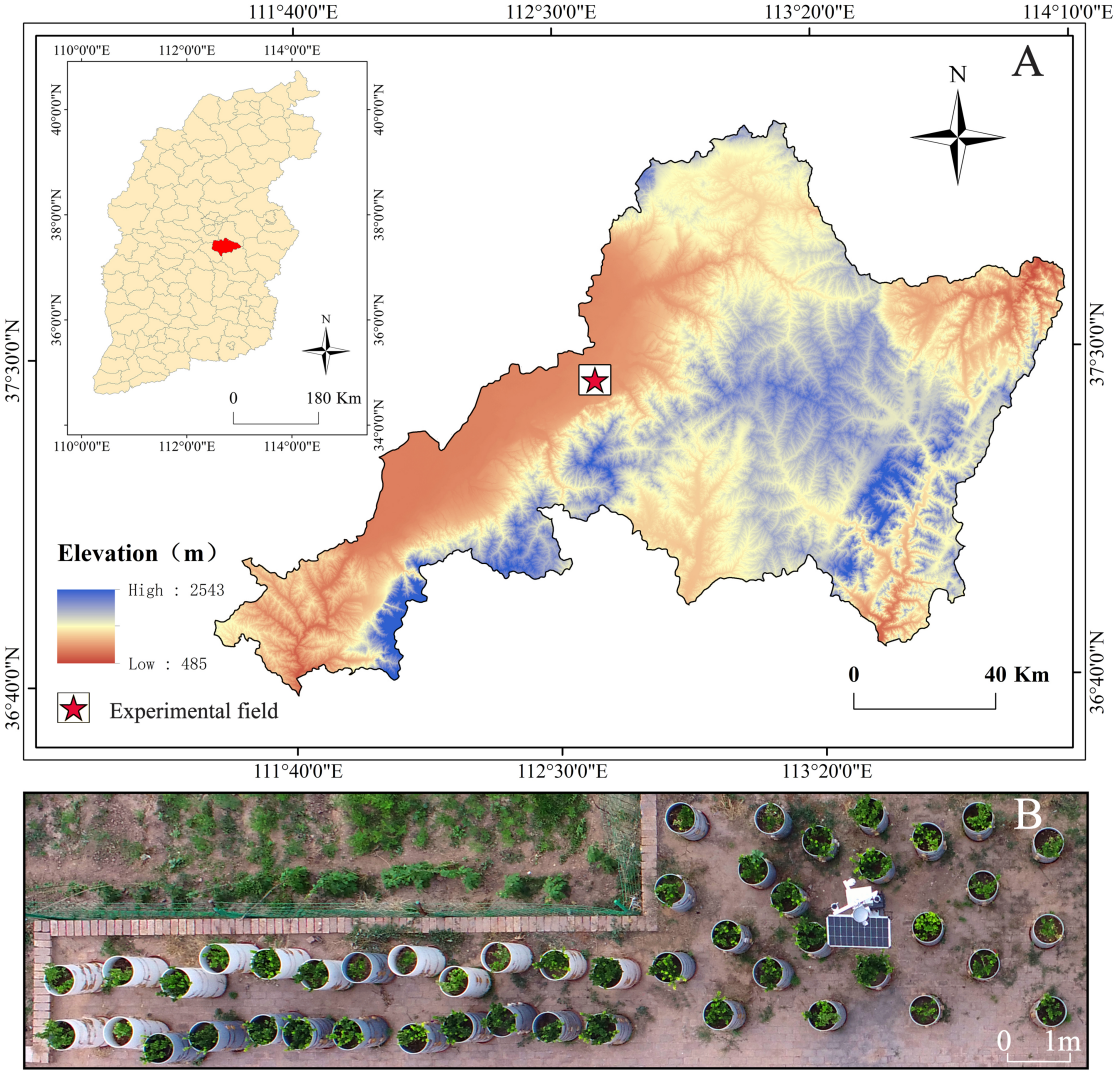
Location of study area. **(A)** The DEM map of Taigu District, Shanxi Province, the pentagram is the location of the experimental field. **(B)** The UAV aerial image of the test field.

The soil used in the study was urban green spaces soil (see Graphical abstract). The experimental site is located in the industry-academia-research training base of the College of Forestry, Shanxi Agricultural University (112°28′–113°01′E, 37°12′–37°3′N), situated in Taigu District, Shanxi Province (Figure 1A). This region lies in the mid-latitude inland Loess Plateau. It belongs to a warm temperate continental semi-arid monsoon climate zone. In July 2023, one-year-old *E. kiautschovicus* plants exhibiting disease-free growth and uniform vigor were selected for potted cultivation (Figure 1B). Before transplantation, aerial parts were pruned to ensure consistent plant height, with three individuals planted per pot. *E. kiautschovicus* is a widely distributed semi-evergreen shrub commonly used in landscaping as hedges and shrub spheres (Yang et al., 2014).

### 2.2 Biochar and compost

The biochar was produced by Henan Lize Environmental Protection Technology Co., Ltd. (China), using corn straw as raw material through a high-temperature pyrolysis process. The corn straw was carbonized at 500–600 °C under oxygen-free constant-temperature combustion for 2 h, resulting in black powdered charcoal after grinding. The compost was sourced from a Japanese organic fertilizer manufacturer, constituting a high-quality microbial fertilizer. Its raw materials (seaweed, fish meal, and soybean meal) underwent pretreatment, primary fermentation, maturation fermentation, and subsequent processing and storage at 60–70 °C. The physicochemical properties of the biochar, compost, and experimental soil are presented in Table 1.

**Table 1:** Basic physical and chemical properties of the modifier and the experimental soil.

Variable	Biochar	Compost	Experimental soil
pH	9.92	8.46	8.40
SOC (g/kg)	510.90	163.49	3.55
TN (g/kg)	8.51	16.59	0.38
TP (g/kg)	2.34	2.73	0.79
${\mathrm{NO}}_{3}^{-}-N$ ‌(mg/kg)	40.57	53.01	12.01
${\mathrm{NH}}_{4}^{+}-N$ (mg/kg)	16.83	27.10	2.76
AP (mg/kg)	11.13	11.31	8.34

### 2.3 Experimental design

The experimental design employed a soil column experiment using PVC (Polyvinyl Chloride) cylinders (100 cm height × 45 cm diameter). Each container was filled with 150 kg of soil to a depth of 90 cm, with the top 30 cm layer amended with varying biochar and compost additions. Three amendment treatments—Control (no amendment), biochar alone, and biochar-compost combinations—were uniformly applied at eight levels based on mass ratios: 0 (CK), 4% biochar (BC4), 8% biochar (BC8), 12% biochar (BC12), 7.5% compost (COM), 7.5% compost + 4% biochar (BCC4), 7.5% compost + 8% biochar (BCC8), and 7.5% compost + 12% biochar (BCC12). The improver was applied to the pot at one time before planting. Sampling was conducted on July 15, 2024, with 0–20 cm depth samples collected from 40 containers across five replicates. All plants were cultivated under identical environmental conditions and standardized management practices.

### 2.4 Analytical methods

Soil pH, temperature, humidity, electrical conductivity (EC), and moisture content were measured and analyzed. Soil pH was determined using a pH meter (PHSJ-3F, Leici, Shanghai, China) with a water-to-soil ratio of 2.5:1. Soil temperature and humidity were measured using a WET-2-KIT soil parameter rapid tester (DELTA-T, UK), while EC was analyzed with a DDS-307A conductivity meter (water-to-soil ratio of 5:1). Soil moisture content was determined by oven-drying at 105 ± 2 °C. Total nitrogen (TN), total phosphorus (TP), and available phosphorus (AP) were quantified using a Martchem450 fully automated discrete chemical analyzer. Available potassium (AK) was measured via ammonium acetate (CH_3_COONH_4_) extraction-flame photometry (Mei, 2019). Microbial biomass carbon (MBC) and nitrogen (MBN) were determined using the chloroform fumigation-K_2_SO_4_ extraction method, while microbial biomass phosphorus (MBP) was analyzed by chloroform extraction-UV spectrophotometry. Soil extracellular enzyme activities were assessed using 96-well microplate fluorometric assays on a multifunctional microplate reader (Fluoroskan Ascent FL, Thermo Scientific) with excitation/emission wavelengths of 365/450 nm. Five key enzymes were measured: β-1,4-glucosidase (BG), cellobiohydrolase (CBH), β-1,4-N-acetylglucosaminidase (NAG), leucine aminopeptidase (LAP), and alkaline phosphatase (AKP) (Gao et al., 2019; Duan et al., 2019). Soil organic carbon (SOC) was determined via potassium dichromate oxidation-spectrophotometry, and easily oxidized organic carbon (EOC) was measured using potassium permanganate oxidation (Lu and Rao, 2025).

### 2.5 Metagenomic sequencing

In July 2024, soil samples from the top layer (0–20 cm) of 40 containers across five replicates were collected for metagenomic sequencing and bioinformatic analysis. Sterilize the sampling equipment and cryotubes using an autoclave, then collect 5 g of homogenized sample from a 20 cm-depth hole in each barrel. Genomic DNA was extracted from soil samples, with concentrations quantified using a Quantus Fluorometer (Picogreen) and integrity assessed via 1% agarose gel electrophoresis. All samples exhibited grade B integrity with partial DNA degradation. DNA was fragmented to 350 bp using Covaris M220, followed by Y-shaped adapter ligation, magnetic bead-based removal of self-ligated adapters, PCR amplification for library enrichment, and NaOH denaturation to generate single-stranded DNA fragments for paired-end (PE) library construction. Shotgun metagenomic sequencing of total microbial genomic DNA was performed on the Illumina high-throughput sequencing platform.

After quality control using fastp to obtain clean data, metagenomic assembly was performed based on contigs ≥1,000 bp. Individual binning was conducted using MetaBAT, CONCOCT, and MaxBin, with DAS_Tool integration to generate metagenome-assembled genomes (MAGs). A two-step dereplication process via dRep included primary clustering at Mash ANI ≥ 90% and secondary clustering at ANI ≥ 99% with ≥10% genome overlap. Medium-quality MAGs (completeness ≥50%, contamination <10%) were selected using CheckM, yielding 182 non-redundant genomes.

### 2.6 Statistical analysis

Statistical analyses were performed using IBM SPSS Statistics 27.0.1. One-way analysis of variance (ANOVA) and Duncan’s multiple range test were applied to assess the effects of different amendment levels on soil physicochemical properties and their significance within the same soil depth. Pearson correlation coefficients were calculated to evaluate inter-variable relationships, with significance tested at *p* < 0.05. Results are expressed as mean ± standard error. Data visualization was conducted using Origin 2021 and Adobe Photoshop 2021. Compositional analysis, comparative analysis, differential analysis, association/module prediction, and MAGs cloud analysis were performed using cloud-based tools from the Majorbio Bio-Cloud Platform.^1^

## 3 Results

### 3.1 Soil physicochemical properties and enzyme activities

**Figure 2:**
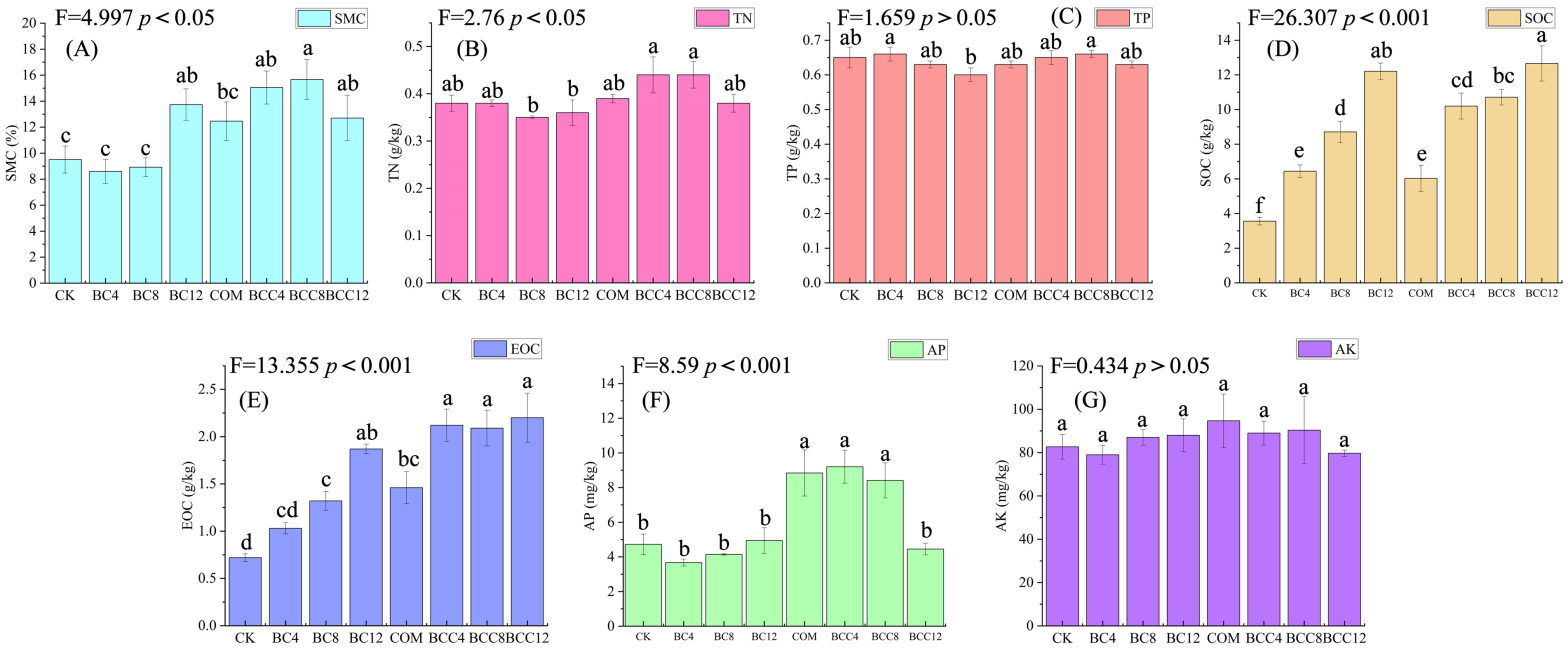
Soil physicochemical properties of different treatments. **(A)** Soil moisture content (SME). **(B)** Total nitrogen (TN). **(C)** Total phosphorus (TP). **(D)** Soil organic carbon (SOC). **(E)** Easily oxidized organic carbon (EOC). **(F)** Available phosphorus (AP). **(G)** Rapidly available potassium (AK). Values are the means ± SE. Lowercase letters indicate significant differences among treatments based on Duncan’s *post hoc* test at the level of *p* < 0.05. F is a statistic used to compare the variance difference between multiple samples. CK, control; BC4, 4% biochar; BC8, 8% biochar; BC12, 12% biochar; COM, 7.5% compost; BCC4, 4% biochar + 7.5% compost; BCC8, 8% biochar + 7.5% compost; BCC12, 12% biochar + 7.5% compost.

BC12 and biochar-compost mixtures significantly improved soil water retention capacity, attributed to the synergistic effects of biochar’s porous structure and organic matter (Figure 2A). For soil nutrient dynamics, compost supplementation increased nitrogen availability, while high-concentration biochar addition restricted nitrogen release (Figure 2B). However, biochar-compost combinations effectively enhanced nitrogen content, with BCC4 and BCC8 showing the highest improvements. Compost elevated AP, but biochar reduced its availability through adsorption and immobilization. COM, BCC4, and BCC8 exhibited higher AP levels compared to CK, with BCC4 reaching peak values (Figure 2F). Compost slightly increased AK, though high biochar concentrations partially offset this effect via adsorption (Figure 2G). SOC and EOC increased with rising biochar concentrations, confirming biochar as the primary contributor to soil carbon content (Figures 2D,E). Medium-low biochar doses combined with compost (BCC4, BCC8) optimized most metrics, whereas high biochar doses (BCC12) reduced efficacy due to adsorption or C/N imbalance. Biochar-driven carbon accumulation directly enhanced SOC through stable carbon inputs, while EOC increased via biochar and compost supplementation. Compost promoted nutrient release, significantly elevating AP, AK, and TN, but required avoidance of high C/N environments. Medium-low biochar doses (4–8%) synergistically improved water retention, carbon pools, and available nutrients when combined with compost, while high doses (12%) induced antagonistic effects via adsorption or C/N imbalance.

**Figure 3:**
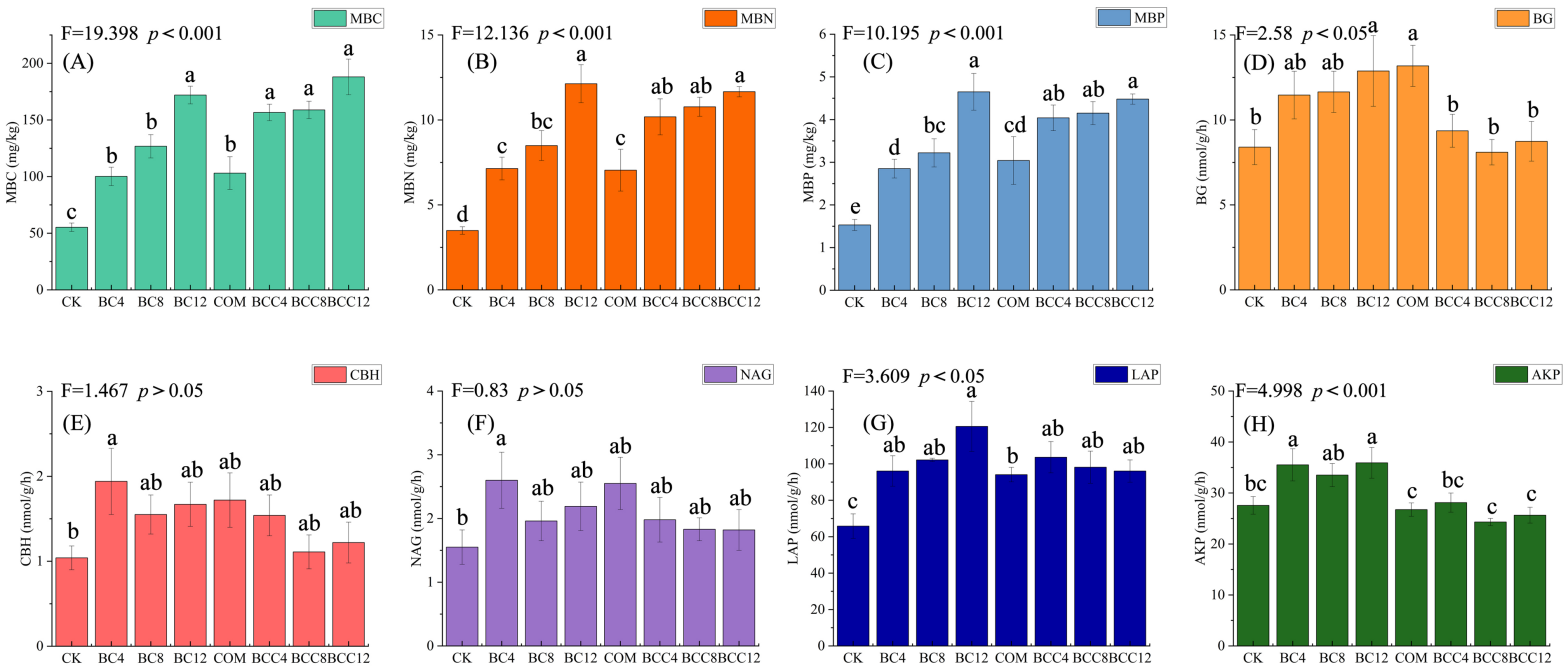
Soil microbial biomass carbon, nitrogen, phosphorus, and their enzyme activities under different treatments. **(A)** Microbial biomass carbon (MBC). **(B)** Microbial biomass nitrogen (MBN). **(C)** Microbial biomass phosphorus (MBP). **(D)** β-1,4-glucosidase (BG). **(E)** Cellobiohydrolase (CBH). **(F)** β-1,4-N-acetylglucosaminidase (NAG). **(G)** Leucine aminopeptidase (LAP). **(H)** Alkaline phosphatase (AKP). Values are the means ± SE. Lowercase letters indicate significant differences among treatments based on Duncan’s post hoc test at the level of *p* < 0.05. F is a statistic used to compare the variance difference between multiple samples. CK, control; BC4, 4% biochar; BC8, 8% biochar; BC12, 12% biochar; COM, 7.5% compost; BCC4, 4% biochar + 7.5% compost; BCC8, 8% biochar + 7.5% compost; BCC12, 12% biochar + 7.5% compost.

MBC increased significantly with rising biochar doses, directly linked to biochar’s high organic carbon content, which supplies stable carbon for microbial proliferation (Figure 3A). MBN increased with biochar concentration due to reduced nitrogen loss via adsorption and enhanced nitrogen-fixing microbial activity under high-carbon conditions (Figure 3B). MBP followed trends similar to MBN, driven by biochar’s phosphorus adsorption and microbial phosphorus immobilization (Figure 3C). COM elevated MBC, MBN, and MBP compared to CK but fell short of high biochar treatments, reflecting compost’s limited total labile organic matter despite rapid decomposition. In combined treatments (BCC4/8/12), medium-low doses (BCC4/8) surpassed single amendments (BC4/8) in MBC, MBN, and MBP, demonstrating synergistic carbon and nutrient supply. High-dose BCC12 achieved peak MBC, MBN, and MBP, but MBN and MBP growth slowed due to nitrogen mineralization inhibition under high C/N ratios.

BG activity increased with biochar concentration, while CBH peaked at low doses, correlating with microbial-driven carbon decomposition demands (Figures 3D,E). Compost alone maximized BG activity due to labile carbon stimulation. Combined treatments generally exhibited lower BG and CBH activities than single amendments, as biochar adsorbed soluble carbon substrates, reducing enzyme induction. Biochar alone caused fluctuating NAG activity but steadily increased LAP, reflecting protease activation under high-carbon conditions for nitrogen acquisition (Figures 3F,G). Compost elevated NAG and LAP activities, maintaining enzyme activity despite direct nitrogen supplementation. Combined treatments showed lower NAG activity than compost alone and slightly higher LAP activity than compost but lower than biochar alone, likely due to biochar-mediated nitrogen adsorption delaying microbial metabolism. AKP activity increased with biochar alone, driven by microbial phosphate secretion in response to phosphorus adsorption (Figure 3H). Compost minimized AKP activity by alleviating phosphorus limitation. Combined treatments exhibited AKP levels near or below CK, as compost reduced phosphorus demand.

### 3.2 Microbial community composition

Based on the non-redundant protein amino acid sequence database NR, species annotation was performed using Diamond v2.0.13 with the Reads Number abundance calculation method. Species abundance was calculated as the sum of gene abundances corresponding to each taxon, yielding four domains (4.32% Archaea, 65.43% Bacteria, 0.14% Eukaryota, and 0.11% Viruses), 13 kingdoms, 239 phyla, 457 classes, 896 orders, 1,815 families, 6,478 genera, and 49,928 species. BCC exhibited the highest values across most taxonomic levels, indicating significant enhancement of microbial taxonomic diversity, particularly at the genus and species levels. BC treatments showed lower values than CK at low concentrations (BC4), with partial recovery at higher concentrations (BC8, BC12) but still failing to surpass CK, suggesting suppressed microbial taxonomic diversity under sole biochar application in *E. kiautschovicus* potted systems. COM slightly exceeded CK at all levels, demonstrating stable but limited improvement compared to high-concentration biochar-compost mixtures.

**Figure 4:**
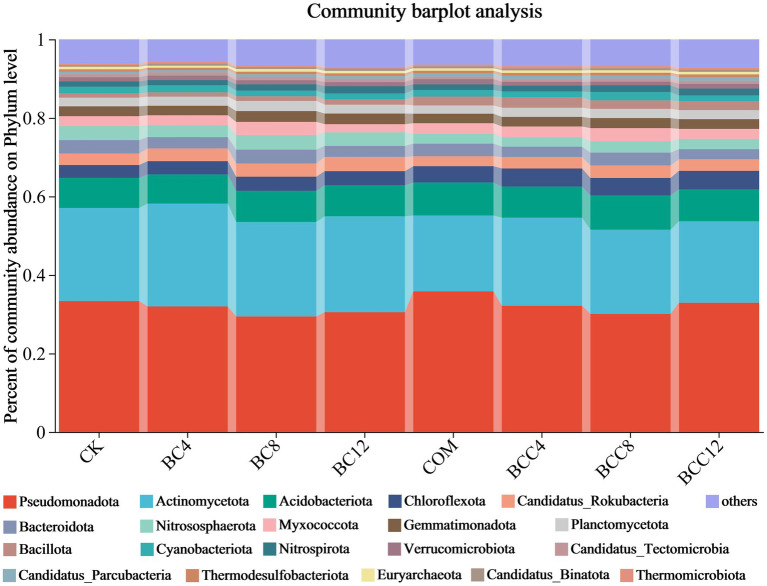
Histogram of species community under different treatments at the phylum level. The community Bar diagram shows the composition of the top 20 species in all samples and the proportion of different species, and other low-abundance species are classified as Others. The abscissa is the sample name, and the ordinate is the proportion of the species in the sample. The columns of different colors represent different species, the square is the species name, and the length of the column represents the proportion of the species. CK, control; BC4, 4% biochar; BC8, 8% biochar; BC12, 12% biochar; COM, 7.5% compost; BCC4, 4% biochar + 7.5% compost; BCC8, 8% biochar + 7.5% compost; BCC12, 12% biochar + 7.5% compost.

At the phylum level (including others), BC application increased Actinomycetota, Nitrospirota, Chloroflexota, and Bacillota. COM alone elevated Pseudomonadota, Bacillota, Acidobacteriota, and Chloroflexota. BCC enhanced Acidobacteriota, Chloroflexota, Bacillota, and Nitrospirota (Figure 4). COM achieved peak Pseudomonadota abundance (35.81%), while BC maximized Actinomycetota levels. BCC8 showed the highest Acidobacteriota abundance (8.72%). Dominant phyla were Pseudomonadota and Actinomycetota. Biochar-driven carbon regulation favored oligotrophic Chloroflexota, which is suitable for long-term soil improvement. Compost enriched copiotrophic Pseudomonadota and Bacillota for short-term fertility enhancement but risked rapid organic matter depletion unless combined with biochar to delay decomposition. Medium-low BCC doses (BCC4/8) balanced carbon input and nutrient release, promoting functional taxa like Chloroflexota and Nitrospirota to support coupled carbon-nitrogen-phosphorus cycling. High carbon load (BCC12) suppressed Pseudomonadota and Actinomycetota activity, potentially hindering plant nutrient acquisition. For enhanced nitrification, prioritize BC12 or BCC8; use COM alone for rapid organic matter decomposition. BCC8 demonstrated optimal integrated effects at the phylum level.

**Figure 5:**
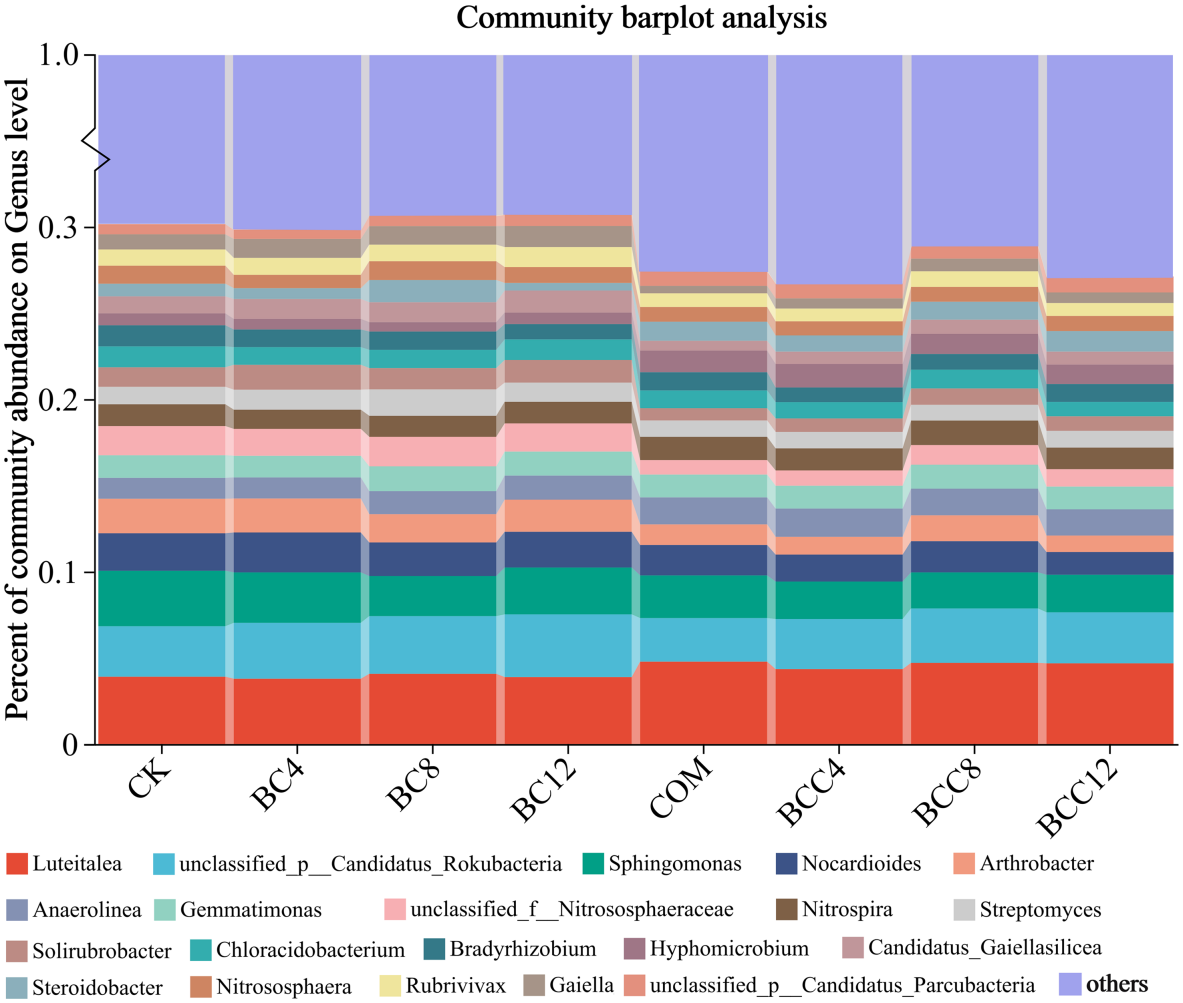
Histogram of species community under different treatments at the genus level. The community Bar diagram shows the composition of the top 20 species in all samples and the proportion of different species, and other low-abundance species are classified as Others. The abscissa is the sample name, and the ordinate is the proportion of the species in the sample. The columns of different colors represent different species, the square is the species name, and the length of the column represents the proportion of the species. CK, control; BC4, 4% biochar; BC8, 8% biochar; BC12, 12% biochar; COM, 7.5% compost; BCC4, 4% biochar + 7.5% compost; BCC8, 8% biochar + 7.5% compost; BCC12, 12% biochar + 7.5% compost.

At the genus level (excluding others), BC alone increased *Streptomyces* and *Gaiella*, while COM elevated *Luteitalea*, *Hyphomicrobium*, and *Anaerolinea* (Figure 5). BCC mixtures enhanced *Luteitalea*, *Nitrospira*, *Anaerolinea*, and *Hyphomicrobium*, but all declined in BCC12. BC8 exhibited superior carbon-nitrogen regulation, with high *Streptomyces* (1.53%) and *Nitrospira* (1.22%) abundances supporting carbon conversion and nitrification. Avoid BC12 due to suppressed *Sphingomonas* (2.72%) and *Bradyrhizobium* (0.89%), impairing pollutant degradation and nitrogen fixation. COM significantly boosted *Luteitalea* (4.78%) and *Hyphomicrobium* (1.25%), suitable for short-term organic decomposition and ammonia oxidation but requiring ammonium nitrogen control. BCC8 synergized *Luteitalea* (4.71%), *Nitrospira* (1.42%), and *Anaerolinea* (1.55%) to balance carbon-nitrogen cycling. BCC12 reduced *Nocardioides* (1.32%) and *Arthrobacter* (0.96%), potentially weakening organic degradation capacity.

Microbial community analysis across phylum and genus levels revealed that high-stability carbon from BC promoted oligotrophic genera (e.g., *Streptomyces*, *Gaiella*), aligning with phylum-level Actinomycetota and order-level Acidimicrobiales responses. Compost-enriched labile carbon favored copiotrophic genera (e.g., *Luteitalea*, *Hyphomicrobium*), reinforcing Pseudomonadota dominance. Combined treatments adsorbed ammonium nitrogen to stimulate *Nitrospira* and *Hyphomicrobium*, consistent with total nitrogen (BCC4 = 0.44 g/kg) and nitrate nitrogen (BC = 40.57 mg/kg) data. Compost’s high ammonium directly stimulated *Hyphomicrobium* (COM = 1.25%) but risked nitrifier inhibition at excessive levels. Compost increased the content of AP (8.84 mg/kg), suppressing AKP’s activity (COM = 26.74 nmol/g/h) while promoting phosphorus-utilizing taxa like Burkholderiales (5.39%). Biochar-adsorbed phosphorus (total P reduced to BC12 = 0.60 g/kg) elevated phosphatase activity (BC12 = 35.92 nmol/g/h) but inhibited phosphorus-sensitive genera (e.g., *Sphingomonas*).

**Figure 6:**
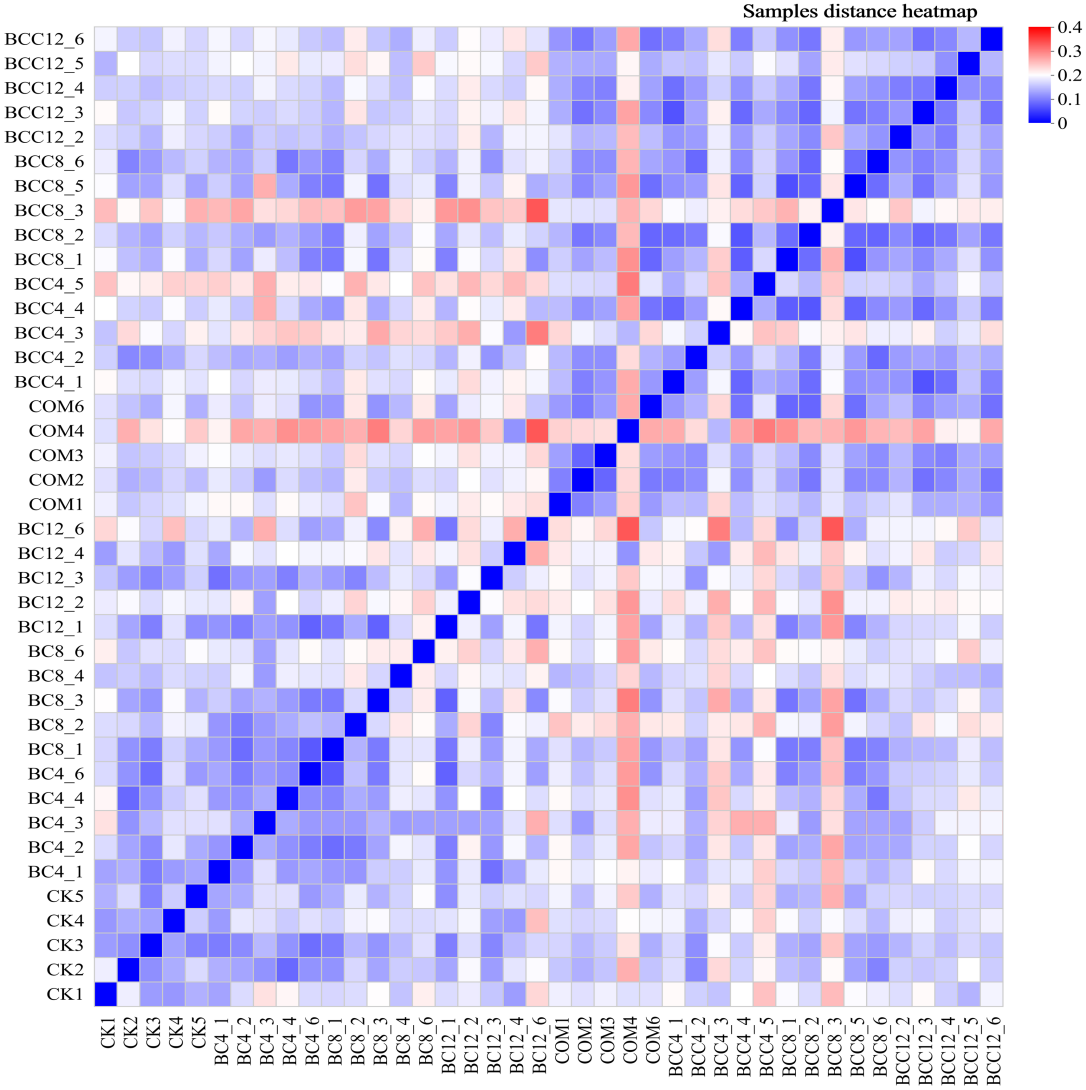
Hierarchical clustering diagram of microbial community samples. In the figure, red indicates that the sample distance is far, and blue indicates that the sample distance is close. CK, control; BC4, 4% biochar; BC8, 8% biochar; BC12, 12% biochar; COM, 7.5% compost; BCC4, 4% biochar + 7.5% compost; BCC8, 8% biochar + 7.5% compost; BCC12, 12% biochar + 7.5% compost. Each gradient has five repeated samples, CK (CK1, CK2, CK3, CK4, CK5).

**Figure 7:**
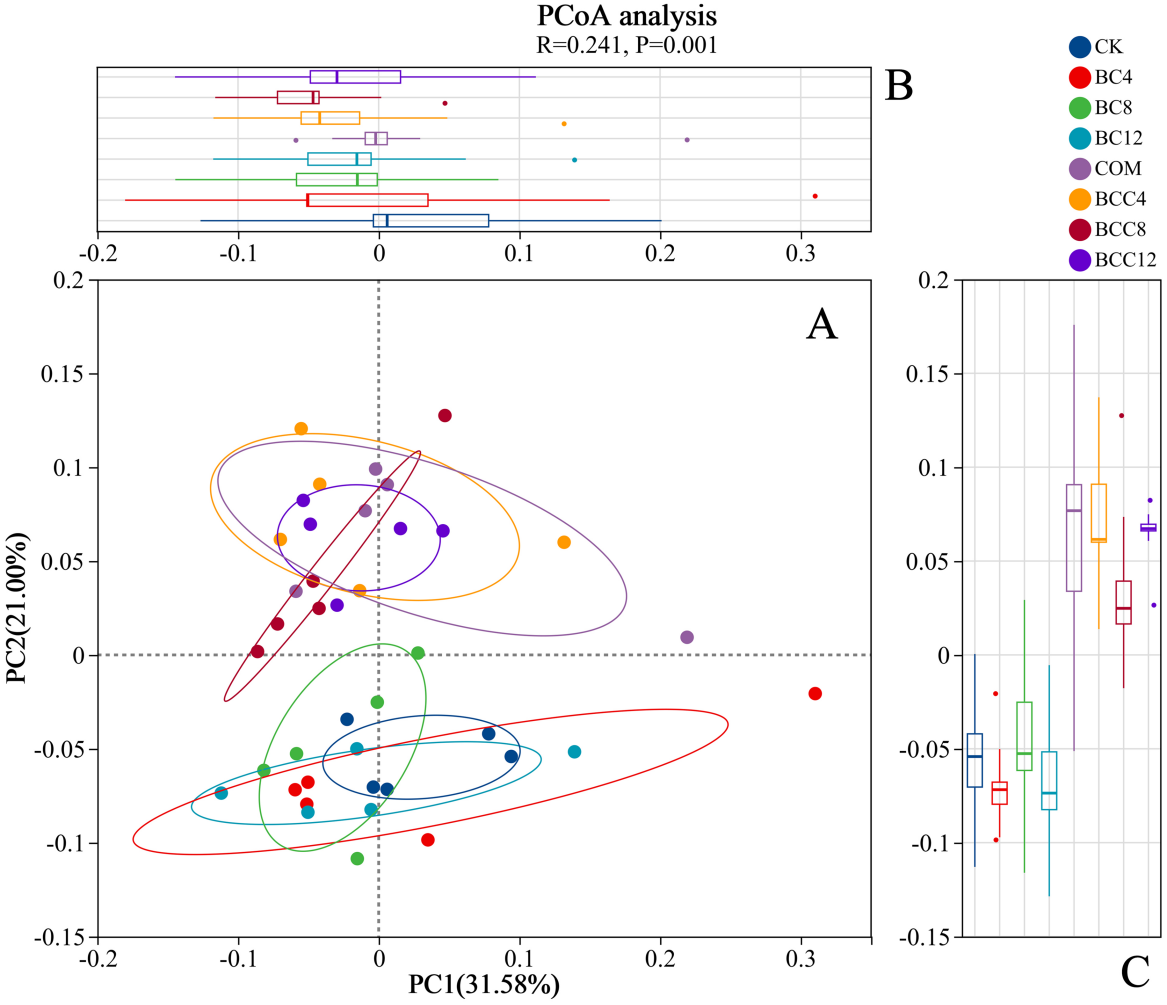
PCoA analysis. **(A)** PC1 and PC2 are two principal coordinate components. PC1 represents the principal coordinate component that explains the data change as much as possible, and PC2 accounts for the largest proportion of the remaining changes. **(B)** PCoA1 axis group difference analysis box diagram. **(C)** PCoA2 axis group difference analysis box diagram. The R value represents the similarity analysis statistic, while the *p* value denotes the significance test index. CK, control; BC4, 4% biochar; BC8, 8% biochar; BC12, 12% biochar; COM, 7.5% compost; BCC4, 4% biochar + 7.5% compost; BCC8, 8% biochar + 7.5% compost; BCC12, 12% biochar + 7.5% compost.

In the hierarchical clustering analysis of order-level microbial communities, with color gradients representing inter-sample distances (red: large distances; blue: small distances). Intra-group distances within CK were minimal (average < 0.15), indicating stable microbial community structures in control groups. Distances between BC treatments and CK increased with rising biochar concentrations (e.g., BC4-CK1: 0.16; BC12-CK1: 0.21), demonstrating that high-dose biochar significantly altered microbial community structures (Figure 6). The smaller distance between BC4 and COM suggested overlapping microbial community structures under low-dose biochar and compost treatments, likely due to synergistic environmental effects. While BC8 exhibited increased distances from CK overall, specific replicates (e.g., BC8_1 and CK3) showed minimal separation (0.092). BC12 displayed the most significant divergence from CK, confirming profound structural shifts induced by BC12. Notably, BC12 and compost treatments exhibited distinct microbial communities, likely due to biochar’s physicochemical-driven selection of adaptive taxa versus compost’s organic matter decomposition-mediated community modulation. Compost significantly altered microbial structures, as evidenced by larger CK-COM distances (e.g., CK1-COM1: 0.1756). PCoA analysis (Figure 7) revealed significant inter-group differences (*p* < 0.05, ANOSIM/PERMANOVA), with CK, COM, and BCC groups showing dispersed distributions. COM and BCC clustered in the PC1 positive direction (0.1–0.3), while CK and BC groups occupied the PC1 negative direction (−0.2–0). Lower PC1 and PC2 values in CK reflected reduced microbial abundance and diversity.

### 3.3 Microbial community diversity

Through Alpha diversity analysis, information on species richness, coverage, and diversity within the community was obtained. The indices sobs, chao, and ace were calculated to reflect community richness, Pielou_e to indicate community evenness, shannon and simpson to assess community diversity, and coverage to evaluate community coverage. The Alpha diversity indices revealed consistent values among sobs, ace, and chao, with a coverage of 1, indicating reliable data. BC4 significantly inhibited richness, evenness, and diversity, potentially associated with microbial stress induced by initial nutrient adsorption or pH alteration caused by biochar. Although the negative effects of BC4 and BC8 diminished, complete recovery was not observed, suggesting either gradual microbial adaptation or saturation of biochar’s adsorption effects. The COM treatment demonstrated superior richness, evenness, and diversity compared to CK, though lower than the BCC series. Composting enhanced microbial growth through organic matter and nutrient supply, yet lacked structural support from biochar. In BCC treatments, biochar’s physical structure provided microbial habitats while compost supplemented nutrients, synergistically enhancing diversity. With increasing biochar concentrations (4% → 12%), BCC treatment efficacy improved progressively. However, BCC12 exhibited slightly lower richness than BCC8, indicating mild inhibitory effects at BC12 concentration.

### 3.4 Soil MAGs and carbon sequestration pathways

#### 3.4.1 MAGs community composition

**Figure 8:**
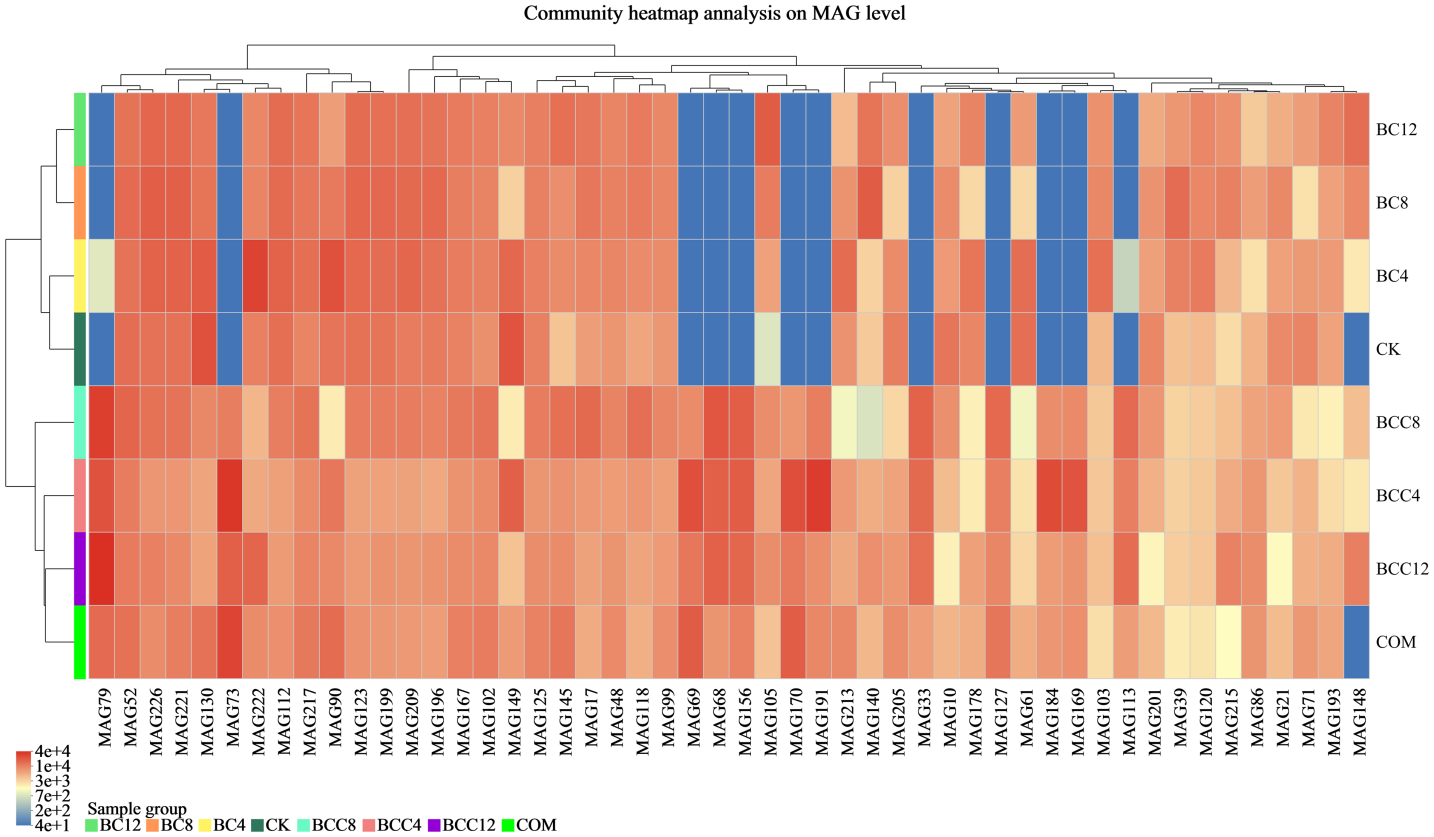
Top 50 MAGs species clustering tree and sample clustering tree. MAGs and the similarity of abundance between samples were clustered. The shades of different colors represent the abundance of species. CK, control; BC4, 4% biochar; BC8, 8% biochar; BC12, 12% biochar; COM, 7.5% compost; BCC4, 4% biochar + 7.5% compost; BCC8, 8% biochar + 7.5% compost; BCC12, 12% biochar + 7.5% compost.

Metagenomic sequences were subjected to binning assembly, and clusters of bins were obtained as MAGs. MAGs with completeness ≥50% and contamination <10% were selected for further analysis. This study reconstructed 182 bacterial and 44 archaeal MAGs, representing predominant species with high abundance in the investigated environment. A species abundance heatmap of the top 50 MAGs was generated, where gradient color intensities in the legend correspond to the relative abundance levels of species (Figure 8).

In the CK group, MAG130 affiliated with *Nitrososphaera* (Thermoproteota), with a completeness of 73.79%; MAG143 affiliated with UBA11222 (Pseudomonadota), with a completeness of 99.78%; and MAG149 affiliated with *Immundisolibacter* (Pseudomonadota), with a completeness of 96.95%, exhibited high abundance. In the COM group, MAG73 (Pseudomonadota, *Methylocaldum*), with a completeness of 98.71%; MAG69 (Pseudomonadota, *Methylocaldum*), with a completeness of 79.36%; and MAG170 (Pseudomonadota, *Methylocaldum*), with a completeness of 85.15%, were highly abundant. In BCC8, MAG79 (Actinomycetota, *ZC4RG35*), with a completeness of 83.76%; MAG68 (Actinomycetota, *ZC4RG17*), with a completeness of 86.15%; and MAG156 (Actinomycetota, *ZC4RG17*), with a completeness of 67.85%, were predominant. The CK and BC groups exhibited lower abundances of MAG79, 73, 69, 68, 156, 170, 191, 33, 127, 184, 169, 113, 186, 30, and 82, whereas the COM and BCC groups demonstrated higher abundance levels, with BCC8 showing the highest overall abundance.

#### 3.4.2 Carbon fixation pathways and key genes encoded by microorganisms

**Figure 9:**
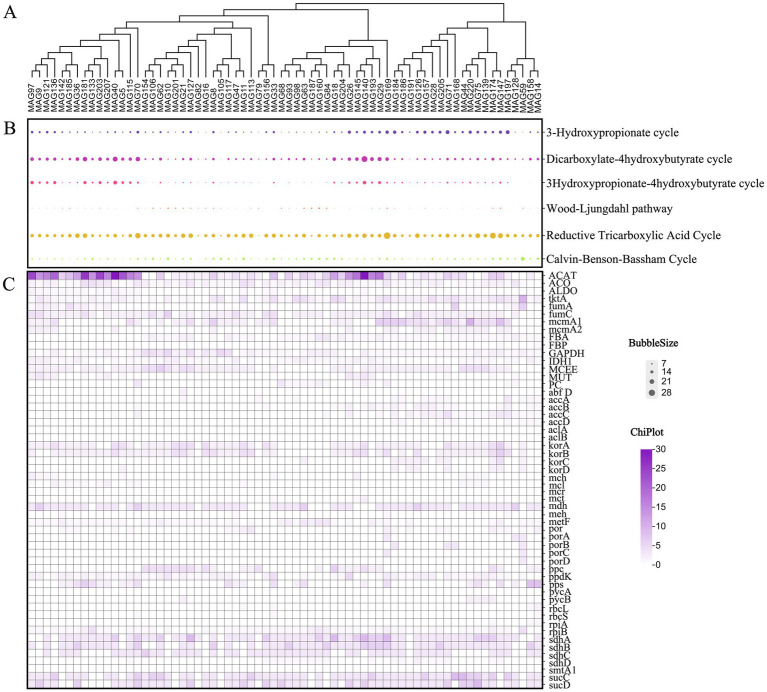
The statistical bubble diagram of the number of genes in the carbon fixation pathway of MAGs and the heat map of carbon fixation genes. **(A)** The main MAGs clustering diagram. **(B)** Six major carbon sequestration pathways. The larger the circle, the more significant the primary carbon cycle corresponding to MAGs is. **(C)** The main carbon fixation genes. The darker the color, the stronger the carbon fixation ability of the carbon fixation gene corresponding to the MAGs.

Analysis of 182 bacterial MAGs revealed six major carbon fixation pathways and their gene distribution patterns (Liu et al., 2017). These pathways include the Reductive Tricarboxylic Acid cycle (rTCA), Dicarboxylate-4hydroxybutyrate cycle (DC/4-HB) cycle, 3-Hydroxypropionate cycle (3-HP), Calvin-Benson-Bassham cycle (CBB), 3Hydroxypropionate-4hydroxybutyrate cycle (3-HP/4-HB), and Wood-Ljungdahl pathway (Figure 9). Based on gene abundance analysis, the six pathways exhibited significant variations across environmental conditions. The rTCA cycle was the most efficient pathway, with an average gene count of 15.03, exemplified by the core MAG176 (34 genes) adapted to high-temperature environments (60 °C). The DC/4-HB cycle ranked second, with an average of 10.95 genes, dominated by MAG115 (26 genes) under anaerobic sulfur-metabolizing conditions. The 3-HP cycle played a critical role in moderately acidic soils (pH 4.5–5.5), averaging 8.91 genes, as seen in MAG29 (17 genes). The CBB cycle, as a universal pathway, was present in all samples (minimum four genes), averaging 6.15 genes, with MAG59 (17 genes) demonstrating high efficiency in carbon fixation. The 3-HP/4-HB cycle and Wood-Ljungdahl pathway served as specialized complementary routes for phototrophic synergy and strict anaerobic environments, averaging 4.95 and 2.71 genes, respectively.

Identification of key microbial groups highlighted MAG176 as a multi-pathway specialist, simultaneously engaged in the rTCA cycle, DC/4-HB cycle, and Wood-Ljungdahl pathway, adapted to extreme thermophilic and sulfur-rich conditions, with elevated abundance in BCC and COM groups. MAG59, specialized in the CBB cycle, emerged as an optimal candidate for agricultural or algal bioreactor applications, showing peak abundance in COM. MAG29 exhibited pathway dominance within the BCC group. Strategic prioritization of rTCA cycle-adapted strains (high-temperature) and DC/4-HB cycle-adapted strains (high-sulfur) is proposed for industrial waste gas treatment and mine remediation, while the CBB cycle serves as a foundational system for light-optimized applications in croplands and photic aquatic environments. These findings provide scientific insights for urban green space carbon management and industrial carbon mitigation, offering novel targets for microbial functional regulation.

Carbon fixation-related genes were curated based on KEGG and literature references (Liu et al., 2017; Peng et al., 2021), encompassing 75 genes (Figure 9). Genes rbcL, rbcS, FBA, GAPDH, and tktA involved in CO_2_ fixation via the CBB cycle were predominantly expressed in MAG105, MAG14, MAG170, and MAG59, correlating with higher CBB cycle activity in BC12, BCC4, and BCC8 treatments (Figures 8, 9). In the rTCA cycle, aclA/B, korA/B, frdA/B, and sdhA/B associated with reductive carboxylation were enriched in MAG176, MAG148, MAG132, and MAG27, corresponding to elevated abundance in BC12, BCC8, and BCC12. Genes por and acsE were enriched in MAG155, MAG14, MAG113, and MAG59, which exhibited active 3-hydroxypropionate cycle activity, potentially driving carbon fixation in this pathway, with peak abundance observed in BCC groups, particularly BCC8.

## 4 Discussion

### 4.1 Synergistic and inhibitory effects of variable dosages

Biochar established a dynamic equilibrium of “microbial biomass enhancement → enzyme activity modulation → nutrient transformation” through carbon provision and nutrient adsorption. Compost directly supplied labile carbon and nutrients, transiently stimulating microbial and enzymatic activity but regulated by biochar adsorption. BCC4/8 balanced carbon inputs and nutrient release, maximizing microbial biomass and enzyme activity. BCC12’s high C/N ratio and adsorption effects induced nitrogen/phosphorus limitation, slowing MBN growth and suppressing AKP, potentially affecting long-term plant growth. Compost alone suits scenarios requiring rapid nutrient availability but requires carbon supplementation to prevent organic matter depletion. Biochar-compost combinations delay organic matter decomposition, synergizing carbon sequestration and nutrient supply. Treatments differentially regulate microbial communities and enzyme activities through carbon stability, nutrient content, and adsorption capacity, ultimately shaping soil nutrient cycling efficiency. Medium-low biochar-compost combinations (BCC4/8) optimize microbial biomass, enzyme activity, and nutrient availability, representing an ideal strategy for *E. kiautschovicus* potted soil management.

Combining low-to-moderate biochar doses (4–8%) with compost (BCC4/8) significantly optimized urban soil physicochemical properties and microbial functionality. Biochar and compost synergistically regulated soil characteristics and enhanced soil utilization efficiency (Figures 2, 3), aligning with previous studies (Biederman and Harpole, 2013; Liang J. et al., 2021; Khaledi et al., 2023). Notably, BCC8 exhibited a marked increase in moisture content compared to CK, consistent with mechanisms by which biochar enhances water retention in Loess Plateau soils (Wang et al., 2018). BCC12 achieved an organic carbon content of 12.66 g/kg, while BCC4 elevated available phosphorus to 9.2 mg/kg, reflecting biochar’s adsorption capacity, stimulation of soil enzyme activity, and mitigation of compost-derived carbon mineralization losses (Li et al., 2025). Moderate biochar addition enhances soil microbial diversity; for instance, 5% biochar altered microbial community structure and increased microbial biomass (Yang et al., 2018). Long-term biochar application elevates bacterial abundance and promotes community diversity (Chen et al., 2018). Biochar addition increased the relative abundance of Bacteroidetes, Actinobacteria, and Acidobacteria while reducing Proteobacteria (Kolton et al., 2011), findings highly consistent with this study. However, high-dose biochar (12%) suppressed Proteobacteria activity by 12% compared to CK due to C/N imbalance and adsorption overload, underscoring the need for precise carbon load regulation in urban soil remediation. In agricultural systems, biochar exceeding 20 t/ha reduces crop yields (Farhangi-Abriz et al., 2021). In contrast, the previous research reported sustained synergism at high doses, potentially due to urban soil constraints like high compaction and low organic matter limiting microbial adaptation (Liu Y. et al., 2022; Han et al., 2023).

### 4.2 Microbial community restructuring and functional responses

**Figure 10:**
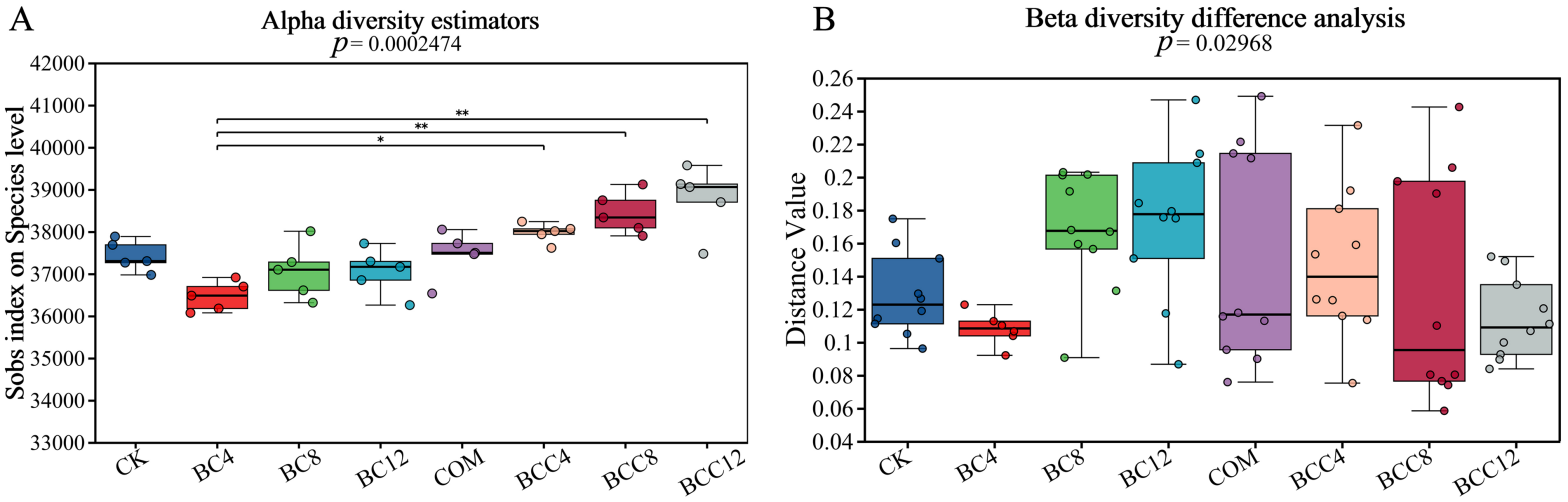
Alpha diversity index box plot and Beta diversity group difference box plot. **(A)** This figure illustrates the significant difference in the index between different groups and the change in the index throughout the experimental study. *p* < 0.05 indicates that the index has a significant difference between the groups. The abscissa is the grouping name, and the ordinate is the index range of each group. **(B)** This analysis presents the significance of community structure differences across various groups. The distance index between samples in different groups is calculated. The points in the box show the distance index between two samples. Based on the distance index between two samples within a group, the difference in community structure between groups is analyzed. *p* < 0.05 indicates that the index has a significant difference between the groups. CK, control; BC4, 4% biochar; BC8, 8% biochar; BC12, 12% biochar; COM, 7.5% compost; BCC4, 4% biochar + 7.5% compost; BCC8, 8% biochar + 7.5% compost; BCC12, 12% biochar + 7.5% compost.

Metagenomic analysis revealed that the combined treatment (BCC8) significantly enriched Acidobacteria (8.72%) and *Nitrospira* (1.42%), driving carbon fixation gene abundance (Figures 4, 5). The enrichment of Acidobacteria, oligotrophic specialists, correlated with stable carbon input from biochar (Kolton et al., 2011), while *Nitrospira* proliferation likely responded to compost-derived ammonium nitrogen (Yao et al., 2017), as COM exhibited 53.01 mg/kg nitrate nitrogen (Table 1). Although biochar addition altered microbial community composition without significantly affecting alpha diversity in maize systems (Hu et al., 2023), this study observed distinct alpha and beta diversity variations across treatments (Figure 10), highlighting differential impacts of biochar dose and compost interactions. Long-term biochar effects on microbial communities were evident in rice paddies 3–4 years post-application (Zheng et al., 2016; Wang et al., 2021); here, shifts occurred within 1 year of single or combined biochar-compost application, with temporal dynamics to be explored in future studies. Proteobacteria dominated compost-only treatments (35.81%) but were suppressed in combined treatments due to biochar adsorption, indicating carbon-nutrient coupling balances copiotrophic and oligotrophic competition.

Biochar’s high stability promoted oligotrophic *Actinobacteria* and *Chloroflexota*, increasing the activities of MBC, AKP, and BG. Compost’s labile carbon enriched copiotrophic Proteobacteria and Flavobacteria, stimulating BG, NAG, and LAP activities to accelerate C/N mineralization. Biochar adsorbed ammonium nitrogen, enhancing Nitrospira and LAP activity, elevating nitrate nitrogen but inhibiting total nitrogen accumulation. Compost directly supplemented ammonium, stimulating *Hyphomicrobium* and *Nitrosomonadales*, though excess ammonium may suppress nitrifiers. Biochar-induced phosphorus adsorption triggered AKP secretion, supporting *Gemmatimonadales* proliferation. Compost moderately increased available phosphorus, suppressing AKP but promoting *Burkholderiales*, a phosphorus-utilizing order. Biochar-compost synergy optimally improved soil physicochemical properties and activity. Low-to-moderate combined doses (BCC4/8) balanced carbon input and nutrient release, fostering functional taxa like *Chloroflexota* and *Nitrospira* to support coupled C-N-P cycling. These findings align with prior studies (Mollick et al., 2020; Kolton et al., 2017; Iqbal et al., 2023; Pandey et al., 2024) while demonstrating unique insights into biochar-compost interactions.

### 4.3 Microbial drivers of carbon fixation pathways

Analysis of MAGs uncovered the distribution patterns of six carbon fixation pathways (Lin et al., 2025). The rTCA cycle, with a mean gene count of 15.03, was dominated by the thermotolerant MAG176, which exhibited higher abundance in BCC and COM groups. The CBB cycle, characterized by broad adaptability (mean gene count: 6.15), was prominently expressed by the photoautotrophic MAG59 in COM, supporting its application in 0-20 cm soil environments, while MAG105, MAG14, and MAG170 showed elevated abundance in BCC4 and BCC8. The 3-hydroxypropionate cycle, driven by MAG27, offers a target for urban green space remediation. The differential distribution of these pathways underscores microbial functional modules’ specificity in responding to environmental factors, providing a basis for targeted regulation. Compared to 16S rRNA analysis, MAGs technology, through whole-genome annotation, enables resolution of microbial functional traits and metabolic potential (Hu et al., 2023), marking the first identification of multi-pathway carbon-fixing consortia in urban soils. MAG176 (Thermoproteota), encoding the rTCA cycle, demonstrated high thermotolerance, while MAG59 (Pseudomonadota), dominant in the CBB cycle, exhibited carbon fixation traits, offering candidate strains for urban carbon sequestration technologies. However, the short-term pot experiment may underestimate long-term biochar-microbe interactions, necessitating subsequent *in situ* monitoring to validate the ecological persistence of functional microbial consortia.

## 5 Conclusion

This study was conducted to quantify the effects of biochar and compost mixing ratio on soil microorganisms and carbon sequestration genes in the urban green spaces. The results showed that BCC4 and BCC8 represent the optimal strategy for urban potted soil remediation, synergistically enhancing water retention, carbon pool stability, and the availability of nutrients. BCC8 notably increased microbial diversity, enzyme activity, and carbon fixation gene abundance. BCC achieved carbon functional balance and enhanced carbon fixation while promoting nutrient cycling. Acidobacteria and Nitrospira emerged as core functional taxa in combined treatments, driving carbon fixation efficiency via rTCA and CBB cycles. The sensitivity of Proteobacteria underscores the need to balance carbon input with nutrient release. Potted *E. kiautschovicus* soil prioritized chemoautotrophic pathways, with the rTCA cycle averaging 15.03 genes per MAG. CBB cycle demonstrated high efficiency in carbon sequestration. These findings highlight the necessity for urban-specific carbon management strategies distinct from agricultural systems and guide the addition method of biochar and compost in the urban green spaces.

## Data Availability

The original contributions presented in the study are publicly available. This data can be found here: https://data.mendeley.com/datasets/2pztvtrv8d/1.

## Footnotes

^1^ https://www.majorbio.com
